# Ocular toxicities associated with targeted anticancer agents: an analysis of clinical data with management suggestions

**DOI:** 10.18632/oncotarget.17634

**Published:** 2017-05-05

**Authors:** Chen Fu, Dan S Gombos, Jared Lee, Goldy C George, Kenneth Hess, Andrew Whyte, David S Hong

**Affiliations:** ^1^ Department of Internal Medicine, New York University Langone Medical Center, NY 10016, USA; ^2^ Department of Internal Medicine, Baylor College of Medicine, TX 77030, USA; ^3^ Department of Phase I Clinical Trials, The University of Texas MD Anderson Cancer Center, TX 77030, USA; ^4^ Department of Biostatistics, The University of Texas MD Anderson Cancer Center, TX 77030, USA; ^5^ Department of Head and Neck Surgery, Division of Ophthalmology, The University of Texas MD Anderson Cancer Center, TX 77030, USA

**Keywords:** targeted, cancer, ocular, toxicity, management

## Abstract

Ocular toxicities are among the most common adverse events resulting from targeted anticancer agents and are becoming increasingly relevant in the management of patients on these agents. The purpose of this study is to provide a framework for management of these challenging toxicities based on objective data from FDA labels and from analysis of the literature. All oncologic drugs approved by the FDA up to March 14, 2015, were screened for inclusion. A total of 16 drugs (12 small-molecule drugs and 4 monoclonal antibodies) were analyzed for ocular toxicity profiles based on evidence of ocular toxicity. Trials cited by FDA labels were retrieved, and a combination search in Medline, Google Scholar, the Cochrane database, and the NIH Clinical Trials Database was conducted. The majority of ocular toxicities reported were low severity, and the most common were conjunctivitis and “visual disturbances.” However, severe events including incidents of blindness, retinal vascular occlusion, and corneal ulceration occurred. The frequency and severity at which ocular toxicities occur merits a more multidisciplinary approach to managing patients with agents that are known to cause ocular issues. We suggest a standardized methodology for referral and surveillance of patients who are potentially at risk of severe ocular toxicity.

## INTRODUCTION

Targeted chemotherapy agents are becoming increasingly important in the clinical management of cancer. Of 42 novel oncologic drugs approved since 2008, 30 were either antibodies or kinase inhibitors targeting specific receptors or unique intracellular signal transduction pathways [1]. This number of approved oncologic drugs is significantly higher than the 15 agents approved between 2000 and 2008. Whereas the toxicity profiles of traditional oncologic agents are known and relatively well-described, the toxicity profiles of targeted therapy agents are not as well-known and include adverse sight-threatening events [2–4]. Ocular toxicities are among the most common adverse events associated with targeted agents [2–7]. This high frequency of ocular adverse events can be partially attributed to the delicate homeostatic environment of growth factors, cell receptors, and vascular formation in the eye, a unique microenvironment that is disrupted by many targeted agents [4,8–13]. Currently, there is a paucity of data documenting particular ocular toxicities of targeted agents [2,4,6]. The purpose of this study is to provide a better understanding of the toxicities that have been observed in various targeted therapies and to provide recommendations for screening, surveillance, and management of these events.

## RESULTS

### Drug selection and FDA label review

Of the 138 agents that were screened for inclusion in the study, 34 were palliative or non-anticancer agents, 12 were duplicates that were approved for separate indications, and 46 were cytotoxic, non-targeted, or conjugated agents and were therefore excluded; the remaining 46 agents were reviewed and screened for their association with ocular toxicities, as described in the Methods section.

FDA labels were reviewed for mention of ocular toxicities, from which 20 agents were associated with some form of ocular toxicity. Three agents (bortezomib, pertuzumab, and dabrafenib) were associated with minor ocular adverse events according to the FDA label, but limited evidence of ocular toxicity was evident upon an independent survey of the literature. Multiple rigorous case reports have associated bortezomib with eyelid chalazia [14–16] and dabrafenib with uveitis and cystoid macula edema [17], however, due to lack of quantified trial data, these agents were excluded from Table 2. Similarly, two agents (idelalisib and ibrutinib) were found to have adverse events from our literature review, but the FDA labels included no mention of ocular toxicity; these agents were excluded from Table 1 but were included in subsequent analysis. A total of 16 agents, including 12 small-molecule drugs and 4 monoclonal antibodies, were analyzed in this study for ocular toxicity profiles based on evidence from FDA labels and clinical trials. Therefore, of the original 46 targeted medications, 18 of 30 small-molecule drugs (60%) and 12 of 16 monoclonal antibodies (75%) were not associated with ocular toxicity and were thus excluded. A summary of ocular toxicities from the FDA label review is included in Table 1.

**Table 1:** FDA label notes regarding ocular adverse events

Therapy	FDA Label Notes
Small-Molecule Targeted Inhibitors	
Afatinib (Gilotrif) [30]	Conjunctivitis 11% n=229 Keratitis 0.8% n=3865 patients with 1 grade III event.
Bortezomib (Velcade) [31]	Label mentions diplopia, blurred vision, conjunctival infection, irritation. Case reports associated with eyelid chalazia.
Ceritinib (Zykadia) [32]	Vision disorder 9% (comprised of vision impairment, blurred vision, photopsia, accommodation disorder, presbyopia, or reduced visual acuity) n=255
Crizotinib (Xalkori) [33]	Vision disorder 64% (Includes diplopia, photopsia, photophobia, vision blurred, visual field defect, visual impairment, vitreous floaters, visual brightness, and visual acuity reduced.) n=255
Dabrafenib (Tafinlar) [34]	Uveitis/Iritis 1% n=586. Can cause cystoid macula edema.
Dasatinib (Sprycel) [35]	Visual disorder (visual disturbance, vision blurred, decreased visual acuity) or dry eye in 1-10% Conjunctivitis in 0.1-1%
Erlotinib (Tarceva) [36]	Reported Conjunctivitis in 18% n=84, Conjunctivitis 12% (with 1 Grade III) n=485 and Keratoconjunctivitis Sicca 12% n=485. Mentions of corneal perforation or ulceration, and abnormal eyelash growth. The pooled incidence of ocular disorders was 17.8% in three lung cancer studies and 12.8% in one pancreatic cancer study.
Gefitinib (Iressa) [37]	Mentions of eye irritation, eye pain, corneal erosion/ulcer, aberrant eyelash growth, corneal membrane sloughing, and ocular ischemia/hemorrhage.
Imatinib (Gleevec) [38]	Estimated 1%-10%: conjunctivitis, vision blurred, eyelid edema, conjunctival hemorrhage, dry eye Estimated 0.1%-1%: eye irritation, eye pain, orbital edema, scleral hemorrhage, retinal hemorrhage, blepharitis, macular edema. Estimated 0.01%-0.1%: papilledema, glaucoma, cataract. Among FDA cited studies (n=729), incidence of periorbital edema was 57.8%, hyperlacrimation 14.1%, and visual disturbance 7.1%.
Nilotinib (Tasigna) [39]	Common: eye hemorrhage, periorbital edema, eye pruritus, conjunctivitis, dry eye. Uncommon: vision impairment, vision blurred, visual acuity reduced, photopsia, eye irritation. Unknown frequency: papilloedema, diplopia, photophobia, eye swelling, blepharitis, eye pain, chorioretinopathy, conjunctival hemorrhage, conjunctivitis allergic, conjunctival hyperaemia, ocular hyperaemia, ocular surface disease, scleral hyperaemia.
Trametinib (Mekinist) [40]	Mentions blurry vision, dry eye, transient blindness, eye floaters, and visual halo. Incidence of Retinal Veinous Occlusion was 0.2% (4/1,749). Incidence of Retinal Pigment Epithelial Detachment was 0.8% (14/1,749).
Vandetanib (Caprelsa) [41]	Blurry vision (corneal opacities) 9%. No cohort information.
Vemurafenib (Zelboraf) [42]	Mentions retinal vein occlusion, uveitis, blurry vision, iritis, and photophobia
Monoclonal Antibodies	
Cetuximab (Erbitux) [43]	Label mentions existence of blepharitis, conjunctivitis, keratitis/ulcerative keratitis with decreased visual acuity, and cheilitis.
Ipilimumab (Vervoy) [44]	Label mentions existence of uveitis, iritis, episcleritis, conjunctivitis, and blepharitis.
Panitimumab (Vectibix) [45]	Eye/eyelid irritation (1%), conjunctivitis (4%), ocular hyperemia (3%), increased lacrimation (2%), in 463 patients
Pertuzumab (Perjeta) [46]	Mentions increased lacrimation (No evidence of this in literature)
Rituximab (Rituxan, Mabthera) [47]	Mentions uveitis and optic neuritis

**Table 2:** Ocular adverse events from studies

Therapy	Total OAEs	Cohort Size	Ocular Adverse Events		
			Most Common	Second Most Common	Other
Small Molecules					
Afatinib (Gilotrif) [48–50]	19	123	Conjunctivitis (NA)	NA	NA
Ceritinib (Zykadia) [51–53]	12	130	Visual Disturbance (12)	NA	NA
Crizotinib (Xalkori) [54,55]	199	322	Visual Disturbance (199)	NA	NA
Dasatinib (Sprycel) [56,57]	6	84	Periorbital Edema (6)	NA	NA
^a^Erlotinib (Tarceva) [58–62]	28	485	NA	NA	NA
Gefitinib (Iressa) [63–66]	170	648	Conjunctivitis (67)	Dry Eyes (35)	Visual Disturbance (29)
Ibrutinib (Imbruvica) [67]	NA	195	Visual Disturbance (19)	Cataract (6)	NA
Imatinib (Gleevec) [68–71]	175	250	Periorbital Edema (175)	Hyperlacrimation (45)	NA
^b^Nilotinib (Tasigna) [71]	NA	556	Periorbital Edema (3)	NA	NA
^a^Trametinib (Mekinist) [72–75]	70	613	NA	NA	NA
Vandetanib (Caprelsa) [76]	21	231	Visual Disturbance (21)	NA	NA
Vemurafenib (Zelboraf) [77,78]	497	3559	Conjunctivitis (131)	Visual Disturbance (82)	Dry Eyes (51)
Total Small Molecules	1197 (19%)	6445			
Monoclonal Antibodies					
Cetuximab (Erbitux) [50]	6	60	Conjunctivitis (5)	NA	NA
Ipilimumab (Vervoy) [18]	37	393	NA	NA	NA
Panitimumab (Vectibix) [79]	61	375	NA	NA	NA
Rituximab (Rituxan, Mabthera) [76,80]	10	132	Conjunctivitis (4)	Visual Disturbance (3)	Periorbital Edema (2)
Total Monoclonal Antibodies	114 (12%)	960			

NA: studies did not report this finding; OAE: ocular adverse event.

^a^Studies did not report individual ocular adverse events.

^b^Studies did not report total ocular adverse events.

### Ocular adverse events

We conducted a review of 217 independent studies, 32 of which met inclusion criteria. The results and ocular adverse events described in these studies are included in Table 2.

Ocular events were scored according to severity and CTCAE grade, as detailed in Table 3. Severe ocular adverse events that were not given a grade were also reported. The most common severe adverse event included severe conjunctivitis, associated with 9 (19.6%) of the total targeted agents, and blurred vision, connected with 10 agents (21.7%). Imatinib had the highest incidence of grade 3 or higher events among all targeted agents, with 3% of patients experiencing grade 3 or higher periorbital edema. Imatinib and crizotinib had the highest incidence of ocular toxicity overall, with 70% and 62-64% of patients experiencing some form of ocular toxicity per FDA statistics and an independent review of available data (Tables 1 and 2). Acute vision-threatening events (including retinal vascular occlusion, retinal pigment epithelial detachment, corneal membrane ulceration and perforation, and blindness) were rare, almost always occurring <1% of the time in their respective drug classes. Only 5 drugs (10.9%) were associated with these vision-threatening events, namely erlotinib, gefitinib, trametinib, vemurafenib, and ipilimumab.

**Table 3:** Severe ocular adverse events (% total patients)

Therapy	Grade I	Grade II	Grade III	Grade IV	Other Serious OAEs
Small Molecules					
Afatinib (Gilotrif) [30,48–50]	Keratitis (0.8%), Conjunctivitis (11%)		Keratitis (0.4%)		
Ceritinib (Zykadia) [32,53]	Vision Disorder (9%)^a^				
Crizotinib (Xalkori) [33,54,55]	Vision Disorder (64%)^b^				
Dasatinib (Sprycel) [35,56,57]	Vision Disorder (7%), Conjunctivitis (<1%)				
Erlotinib (Tarceva) [36,58–62]	Conjunctivitis (5.3%), Keratoconjunctivitis Sicca (2.5%)		Conjunctivitis (<1%)		Corneal Perforation or Ulceration
Gefitinib (Iressa) [37,63–66]	Conjunctivitis (7%), Dry Eyes (8%), Corneal Erosion (0.5%), Visual Disturbance (4%)^c^, Superficial Punctate Keratopathy (0.2%)	Dry Eye (<1%), Conjunctivitis (1%), Corneal Erosion (<1%), Superficial Punctate Keratopathy (<1%)	Corneal Erosion (<1%)		Corneal Ulcer, Corneal Membrane Sloughing, Ocular Ischemia and Hemorrhage
Ibrutinib (Imbruvica) [67,81]	Visual disturbance (10%), Cataract (3%)				
Imatinib (Gleevec) [38,68–71]	Periorbital edema (57.8%)	Periorbital edema (21.2%)			
	Conjunctivitis (13%),		Periorbital Edema (3%), Visual Disturbance (0.8%)		
Nilotinib (Tasigna) [39,71]	Periorbital Edema (1%)		Periorbital Edema (0.2%)		Eye Hemorrhage, Visual Impairment
Trametinib (Mekinist) [40,72–75]	Dry eye (2%), Blurred vision (2%)		Central Serous Chorio-retinopathy Figure 2 (0.5%)		Retinal Veinous Occlusion Figure 4 (0.2%), Retinal Pigment Epithelial Detachment (0.8%)
Vandetanib (Caprelsa) [41,76]	Corneal Opacities (9%)				
Vemurafenib (Zelboraf) [42,77,78]	Blurred Vision (2.4%)^d^, Conjunctivitis (4%), Eye Pain (0.9%), Uveitis (1.3%)		Uveitis (0.2%), Cataracts (0.1%), Iridocyclitis (0.1%), Reduced visual activity (<0.1%)	Uveitis (<0.1%), Amaurosis (<0.1%), Macular Edema (<0.1%), Retinal Artery Occlusion (<0.1%), Conjunctivitis (<0.1%)	Macular Degeneration, Blepharitis, Glaucoma, Papilledema, Retinal Detachment, Vitreous Hemorrhage
Monoclonal Antibodies					
Cetuximab (Erbitux) [43,50]	Conjunctivitis (1-10%)		Conjunctivitis (<1%)		
Ipilimumab (Vervoy) [18,44]			Cataract (<1%), Uveitis (<1%)	Blindness (<1%)	Uveitis, Iritis, Episcleritis, Graves-like Ophthal-mopathy
Panitimumab (Vectibix) [45,79]	Conjunctivitis (6%), Ocular Hyperemia (3%), Hyperlacrimation (2%)				
Rituximab (Rituxan, Mabthera) [47,76,80]	Conjunctivitis (3%), Visual Disturbance (2%), Eye Pain (1%)		Glaucoma (<1%)		

OAE: ocular adverse event.

^a^Comprises vision impairment, blurred vision, photopsia, accommodation disorder, presbyopia, or reduced visual acuity.

^b^Includes diplopia, photopsia, photophobia, vision blurred, visual field defect, visual impairment, vitreous floaters, visual brightness, and reduced visual acuity.

^c^Includes blurred vision, bilateral hemianopia, and photophobia.

^d^Of the total visual disturbances (78/3222), 41 were blurred vision, 22 vision impairment, 12 reduced visual acuity, 2 transient blindness, 1 blindness.

The most severe ocular adverse events occurred with EGFR, MEK and CTLA-4 inhibitors and targeted antibodies. These events included corneal perforation and retinal vascular occlusion. Retinal vascular occlusion had an incidence of 0.8%. Corneal perforation was described primarily in case reports and case studies; however, 3 patients in a large phase 1 trial of gefitinib with a cohort of 221 patients experience grades 1-3 corneal erosion and corneal defects. Use of ipilimumab resulted in the most severe adverse event, causing blindness in 2 out of 393 patients [18].

Small-molecule drugs appear to have a higher incidence of ocular toxicity than do monoclonal antibodies. The percentage of small-molecule drugs associated with ocular toxicity (37.5%) was higher than that associated with monoclonal antibodies (28.6%). Even within the same class of action, monoclonal antibodies resulted in fewer adverse events than did their small-molecule counterparts.

### Summary and management recommendations

A summary of ocular events and management recommendations are presented in Table 4. While there is a paucity of data on this subject, our recommendations are based on available data from clinical trials, case reports and series, clinical experience, expert opinion, and existing national guidelines. One of the central underlying themes in the management recommendations is the concept of establishing a pretreatment baseline to better gauge subsequent ocular adverse events. Screening suggestions were modeled after preferred practice patterns on the management of ocular toxicities of hydroxychloroquine, issued by the American Academy of Ophthalmology, [19] and of ethambutol, issued by the Hong Kong Ophthalmologic Society [20]. Guidelines were referenced according to frequency of screening in drugs that showed a similar incidence and severity of ocular toxicities. From this, specific screening parameters were formulated based on severity of adverse events and on drug mechanism. For instance, screening suggestions for MEK inhibitors focused on assessing retinal integrity due to MEK inhibition's potent effect on retinal maintenance and repair, as evidenced by its severe adverse event profile.

**Table 4:** Common and serious ocular adverse events with management recommendations

Therapy	Common Adverse Events	Serious Adverse Events	Management Recommendations (From DSG)
Small Molecules			
EGFR Inhibitors (gefitinib, erlotinib, afatinib)	Conjunctivitis, Blepharitis, Trichomegaly	Corneal Ulceration or Perforation, Ocular Ischemia	Screening: Recommend pre-treatment ophthalmic exam including slit lamp exam and dilated fundoscopic exam. Annual screening may be conducted for asymptomatic corneal signs including redness or pain. Reassess contact lens-wear. Management: Cases of Conjunctivitis have primarily been grade 2 or lower, with one grade 3 event. Withhold drug for up to 14 days with evidence of corneal abnormalities.
Mixed VEGFR/EGFR Inhibitors (vandetanib)	Corneal Opacities	NA	Screening: Recommend pre-treatment ophthalmic exam including slit lamp exam and dilated fundoscopic exam. Management: Refer at baseline prior to initiation of medication to assess for any underlying corneal pathology. Stop medication and refer to ophthalmologist immediately if patient develops any ocular symptoms. Topical steroids may be necessary but should only be administered by an ophthalmologist
Tyrosine Kinase Inhibitors (imatinib, nilotinib, dasatinib)	Periorbital Edema, Hyperlacrimation, Subconjunctival Hemorrhage	NA	Screening: Recommend pre-treatment ophthalmic exam including slit lamp exam and dilated fundoscopic exam. Management: Periorbital edema continues to be a common event with tyrosine kinase inhibitors, namely with imatinib. In severe cases, surgical debulking has improved symptoms considerably. Consider ophthalmology referral for severe cases of periorbital edema
MEK Inhibitors (trametinib)	Visual Disturbances Including Transient Blindness	Retinal Vein Occlusion, Central Serious Chorioretinopathy or Retinal Pigment Epithelial Detachment	Screening: Recommend pre-treatment ophthalmic exam including slit lamp exam and dilated fundoscopic exam. Patient screening for risk factors attributed to the development of severe events (e.g., hypertension, CAD, baseline visual deficits) should be considered before administration of MEK inhibitors. Baseline OCT, fundus photography, and Amsler grid may be performed to identify macular problems. Eye exams may be conducted every 3-6 months to monitor for severe OAEs. Management: The drug should be discontinued with signs of any serious ocular events and an ophthalmologist should be consulted. FDA label recommends holding drug for up to 3 weeks with signs of grade 2-3 retinal pigment epithelial detachment. Do not modify dabrafenib if used in combination. It is commonly believed that subretinal fluid associated with MEK inhibitors will resolve even with continuation of the drug.
ALK Inhibitors (ceritinib, crizotinib)	Visual Disturbances (Photopsia and Trailing Lights)	NA	Screening: Recommend pretreatment ophthalmic exam including slit lamp exam and dilated fundoscopic exam.
BTK Inhibitors (ibrutinib)	Visual Disturbances	NA	Screening: Recommend pre-treatment ophthalmic exam including slit lamp exam and dilated fundoscopic exam.
V600E Mutated BRAF Inhibitors (vemurafenib)	Conjunctivitis, Cystoid Macular Edema	Uveitis, Amaurosis, Retinal Artery Occlusion	Screening: Recommend pre-treatment ophthalmic exam including slit lamp exam and dilated fundoscopic exam. Patient screening for risk factors attributed to the development of severe events (e.g., hypertension, CAD, baseline visual deficits) should be considered before administration of vemurafenib, especially in conjunction with MEK inhibitors. Eye exams may be conducted every 3-6 months to monitor for more severe ocular adverse events. Management: The drug should be discontinued with signs of any serious ocular adverse events and an ophthalmologist should be consulted.
Monoclonal Antibodies			
Anti-EGFR Antibodies (cetuximab, panitumumab)	Conjunctivitis	NA	Screening: Recommend pre-treatment ophthalmic exam including slit lamp exam and dilated fundoscopic exam. .
Anti-CTLA4 Antibodies (ipilimumab)	NA	Episcleritis, Blindness	Screening: Recommend pre-treatment ophthalmic exam including slit lamp exam and dilated fundoscopic exam. Screen for history of risk factors including history of ocular inflammatory disease and temporal arteritis. Management: Known cases of blindness occurred secondary to Posterior Reversible Encephalopathy Syndrome and were reversed with treatment of PRES. As such risk factors for the development of blindness or PRES should be considered before administration of the drug. FDA Label states: Permanently discontinue YERVOY for clinically significant or severe immune-mediated adverse reactions. Initiate systemic corticosteroids at a dose of 1 to 2 mg/kg/day prednisone or equivalent for severe immune-mediated adverse reactions. Administer corticosteroid eye drops to patients who develop uveitis, iritis, or episcleritis. Permanently discontinue YERVOY for immune-mediated ocular disease that is unresponsive to local immunosuppressive therapy.
Anti-CD20 Antibodies (rituximab)	Conjunctivitis	NA	Screening: Recommend pre-treatment ophthalmic exam including slit lamp exam and dilated fundoscopic exam.

## DISCUSSION

Ocular toxicities are becoming increasingly relevant with the increased use of targeted agents. Sparse data exist with regard to specific recommendations for the screening and management of these toxicities. The goal of this study was to review data from FDA labels and from independent studies to determine which agents need ophthalmologic management and to provide recommendations for the screening and management of patients receiving these agents. Management recommendations have already been explored by various groups. The FDA labels include detailed recommendations for at least two agents, namely trametinib and ipilimumab. These recommendations are incorporated into this study. Van der Noll et al. presented a review of ocular toxicity with management recommendations and algorithms for serous retinal detachment and retinal veinous occlusion in 2013 [21]. Our study provides a comprehensive and agent-specific set of recommendations for the management and screening of known ocular toxicities.

Our data analysis showed that for the majority of agents, the most common types of ocular toxicity were low-grade in severity, primarily grade 1 or 2 according to the CTCAE [22]. Table 5 provides a detailed list of adverse events and the agents that cause them. The most common complications among all agents (both small-molecules agents and monoclonal antibodies) were conjunctivitis and “visual disturbances.” However, the progression of even the most common toxicities was not consistent, and some toxicities were severe, as evidenced by at least two case reports in which rapid progression to blindness occurred with use of ipilimumab and crizotinib [18,23]. As such, the establishment of a visual baseline is important to assess the severity and progression of signs and symptoms that may arise throughout the course of management. We believe that the incidence of ocular toxicities in certain agents and the potential for severity is enough to merit ophthalmic referral when prescribing these agents. The most important suggestion based on these data is the establishment of an ophthalmic baseline for targeted anticancer drugs known to cause ocular toxicities.

**Table 5:** Adverse events and their causes

Adverse Event	Causes	Management Recommendations for the Medical Oncologist (From DSG)
Amaurosis	Vemurafenib	Immediate assessment by ophthalmology. Withhold drug until seen by an ophthalmologist.
Blepharitis	Gefitinib, Erlotinib, Afatanib	Prior to therapy refer for baseline assessment to ophthalmologist if patient known to have blepharitis. If there is baseline blepharitis that is mild to moderate supportive care with lid scrubs should be initiated. If blepharitis progresses consider adding a topical ophthalmic antibiotic ointment to the lid margin such as erythromycin or bacitracin.
Cataract	Ibrutinib, Ipilimumab	Continue management. Refer to ophthalmology regarding assessment for cataract surgery.
Central Serous Chorioretinopathy (Figure 4)	Trametinib	Discontinue drug until assessment by an ophthalmologist. Consider dose modification or cessation of drug depending on ophthalmologist recommendations.
Conjunctivitis	Afatinib, Dasatinib, Erlotinib, Gefitinib, Imatinib, Vemurafenib, Cetuximab, Panitimumab, Rituximab	Continue management. Consider subacute assessment within 1 month by ophthalmologist.
Corneal Erosion or Abrasion	Erlotinib, Gefitinib	Immediate assessment by ophthalmology. Withhold drug until seen by an ophthalmologist.
Corneal Membrane Sloughing	Gefitinib	Immediate assessment by ophthalmology. Withhold drug until seen by an ophthalmologist.
Corneal Opacities	Vandetanib	Refer at baseline prior to initiation of medication to assess for any underlying corneal pathology. Stop medication and refer to ophthalmologist immediately if patient develops any ocular symptoms. Topical steroids may be necessary but should only be administered by an ophthalmologist
Corneal Perforation	Erlotinib	Immediate referral to Ophthalmology. Consider withholding drug or modifying drug dosage until assessment by an ophthalmologist
Corneal Ulceration	Erlotinib, Gefitinib	Immediate assessment by ophthalmology. Withhold drug until seen by an ophthalmologist
Cystoid Macular Edema	Vemurafenib, Dabrafenib	Immediate referral to ophthalmologist (1-3 days). If confirmed stop drug and see if edema resolves. If edema resolves can consider resuming drug at lower dose or change drug. If edema does not resolve refer to retina specialists consider local intravitreal therapy
Dry Eyes	Gefitinib, Trametinib	Initiate artificial tears. Consult ophthalmology and consider continuing drug or modifying the dose upon consultation
Episcleritis	Ipilimumab	Subacute assessment within 1 month by ophthalmology. Consider withholding drug until ophthalmology consult.
Eye Pain	Vemurafenib, Rituximab	Subacute assessment within 1 month by ophthalmology. Consider withholding drug until ophthalmology consult.
Glaucoma	Rituximab	Immediate assessment by an ophthalmologist is merited with signs and symptoms of acute angle closure glaucoma. Otherwise, consider subacute referral within 1 month to ophthalmology
Graves Like Ophthalmopathy	Ipilimumab	Ophthalmology assessment within 2-3 weeks. Consider dose alteration of withholding medications until assessment by an ophthalmologist
Hyperlacrimation	Panitimumab	Continue medical regimen. Routine ophthalmology referral.
Iridocyclitis/Iritis	Vemurafenib, Ipilimumab	Ophthalmology assessment within 1-2 weeks. Consider withholding medication until ophthalmology assessment.
Keratitis	Keratitis	If mild, initiate artificial tears, refer for ophthalmology assessment within 1-2 weeks and continue drug. If severe, immediate referral to ophthalmology is merited with considerations to withhold the medication until assessment.
Keratoconjunctivitis Sicca	Erlotinib	If mild, refer for ophthalmology assessment within 1-2 weeks and continue drug. If severe, immediate referral to ophthalmology is merited with considerations to withhold the medication until assessment.
Macular Edema	Vemurafenib	If severe, refer for immediate assessment by ophthalmology and consider withholding medication until assessment.
Ocular Hemorrhage	Gefitinib	If severe, refer for immediate assessment by ophthalmology and consider withholding medication until assessment.
Ocular Ischemia	Gefitinib	If severe, refer for immediate assessment by ophthalmology and consider withholding medication until assessment.
Periorbital Edema	Imatinib, Nilotinib,	If mild, refer for ophthalmology assessment within 1-2 weeks and continue drug. If severe, immediate referral to ophthalmology is merited with considerations to withhold the medication until assessment.
Retinal Artery Occlusion	Vemurafenib	Withhold medication and refer for immediate assessment by an ophthalmologist if symptoms have occurred within a 24-hour timespan. If symptoms have persisted for greater than 24 hours, subacute referral to ophthalmology is merited.
Retinal Pigment Epithelium Detachment	Trametinib	Refer for immediate assessment by an ophthalmologist. Consider dose modification or withholding the medications until ophthalmology assessment.
Retinal Venous Occlusion (Figure 1)	Trametinib	Withhold medication and refer for immediate assessment by an ophthalmologist if symptoms have occurred within a 24-hour timespan. If symptoms have persisted for greater than 24 hours, subacute referral to ophthalmology is merited
Subconjunctival Hemorrhage	Imatinib, Nilotinib	Continue drug. Routine referral to ophthalmologist not acute. Supportive care as needed
Superficial Punctate Keratopathy	Gefitinib	If mild, initiate artificial tears, refer for ophthalmology assessment within 1-2 weeks and continue drug. If severe, immediate referral to ophthalmology is merited with considerations to withhold the medication until assessment.
Trichomegaly	Gefitinib, Erlotinib, Afatanib	Trim lashes as needed. Refer to ophthalmologist if symptoms affect vision or if direct lash/corneal touch develops
Uveitis	Ipilimumab, Vemurafenib, Dabrafenib	If mild, consider initiating topical steroids, holding medication or dose modification, and subacute ophthalmology assessment within 1 month. If severe, hold medication and refer for immediate assessment by an ophthalmologist.
Visual Disturbance	Ceritinib, Crizotinib, Dasatinib, Gefitinib, Ibrutinib, Imatinib, Nilotinib, Trametinib, Vemurafenib, Rituximab	Management depends on severity. Consider ophthalmic consultation especially with drugs with known sight threatening events (e.g., trametinib, crizotinib, imatinib).

**Figure 1:**
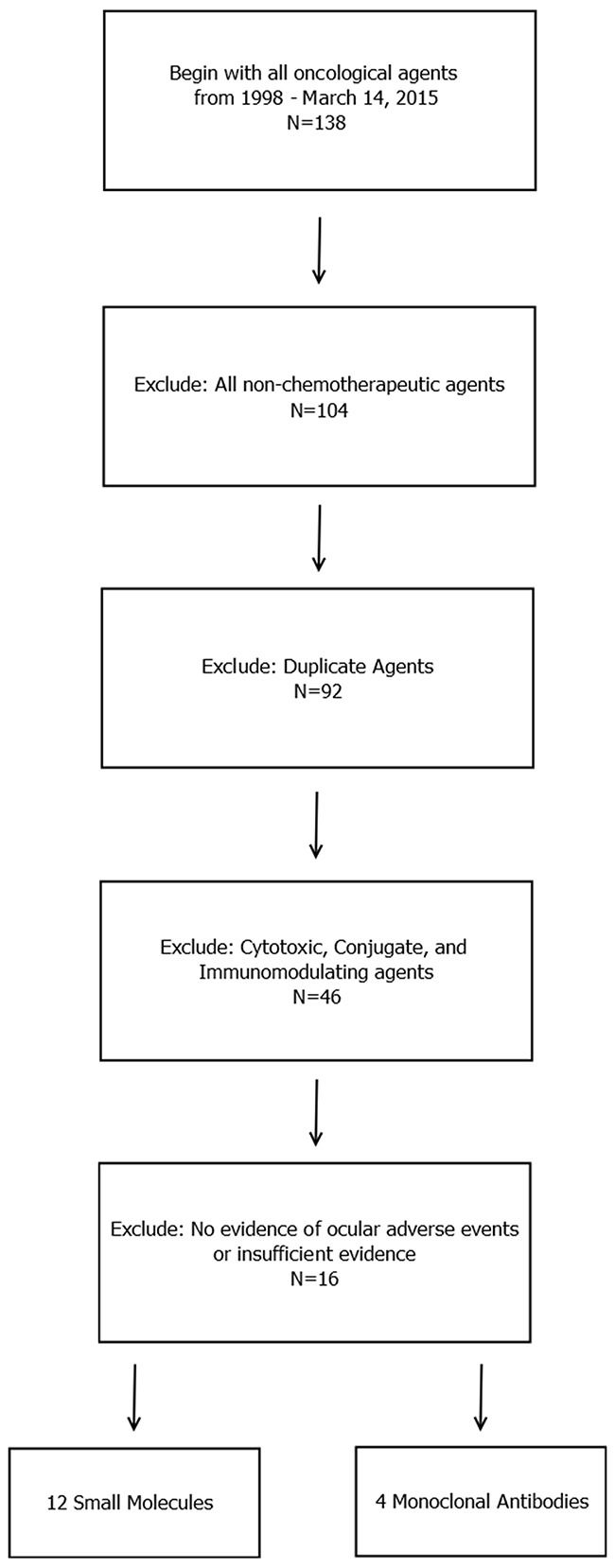
Fundus photograph of the left eye in a patient who developed grade 1 branch vein occlusion while undergoing MEK inhibitor therapy Arrows denote dot blot hemorrhages in the fundus, consistent with occlusion.

**Figure 2:**
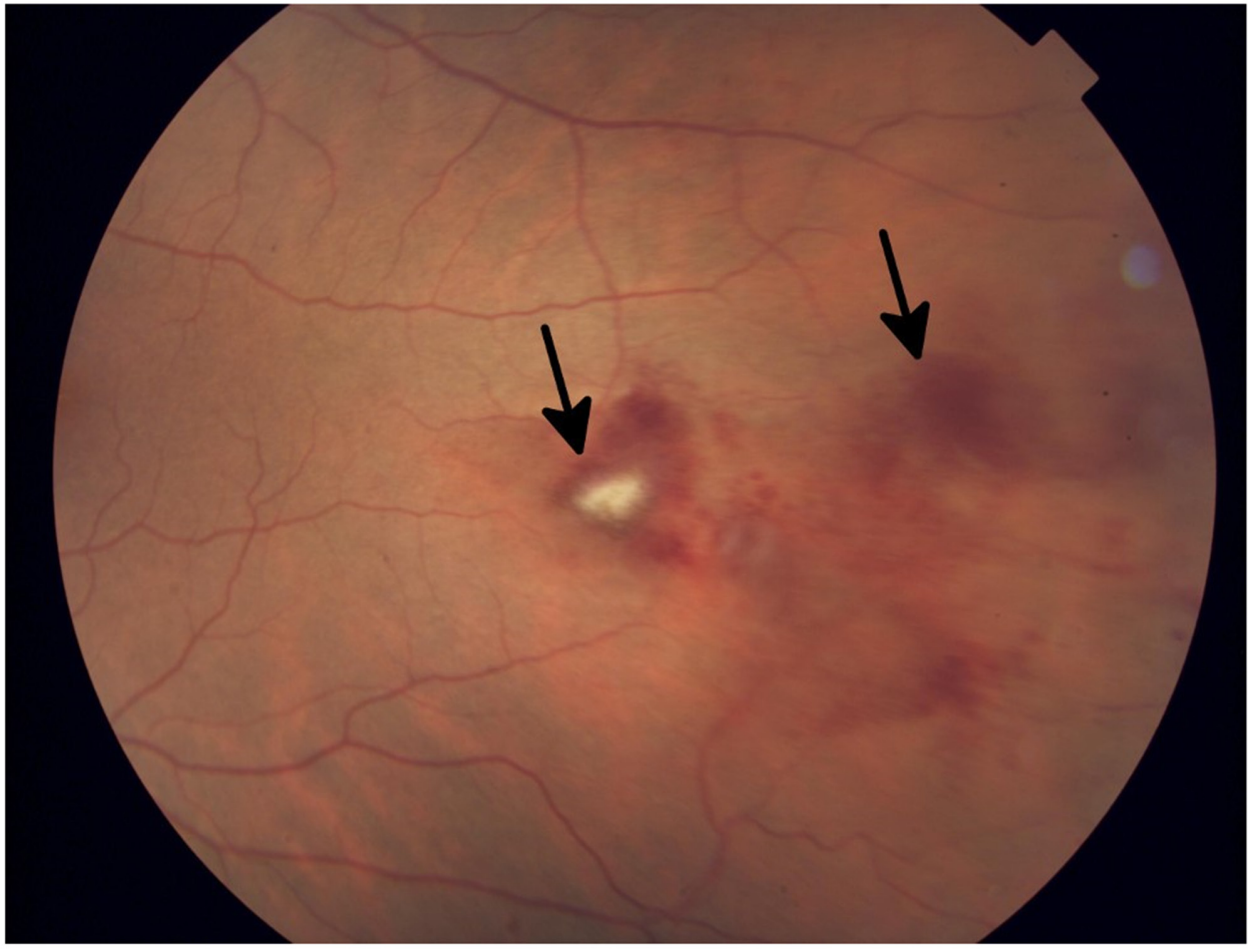
Slit lamp photograph of grade 3 diffuse microcystic changes (arrow) in a patient undergoing treatment with an EGFR inhibitor The patient subsequently developed ocular hypertension due to the topical steroid used to treat the microcysts.

**Figure 3:**
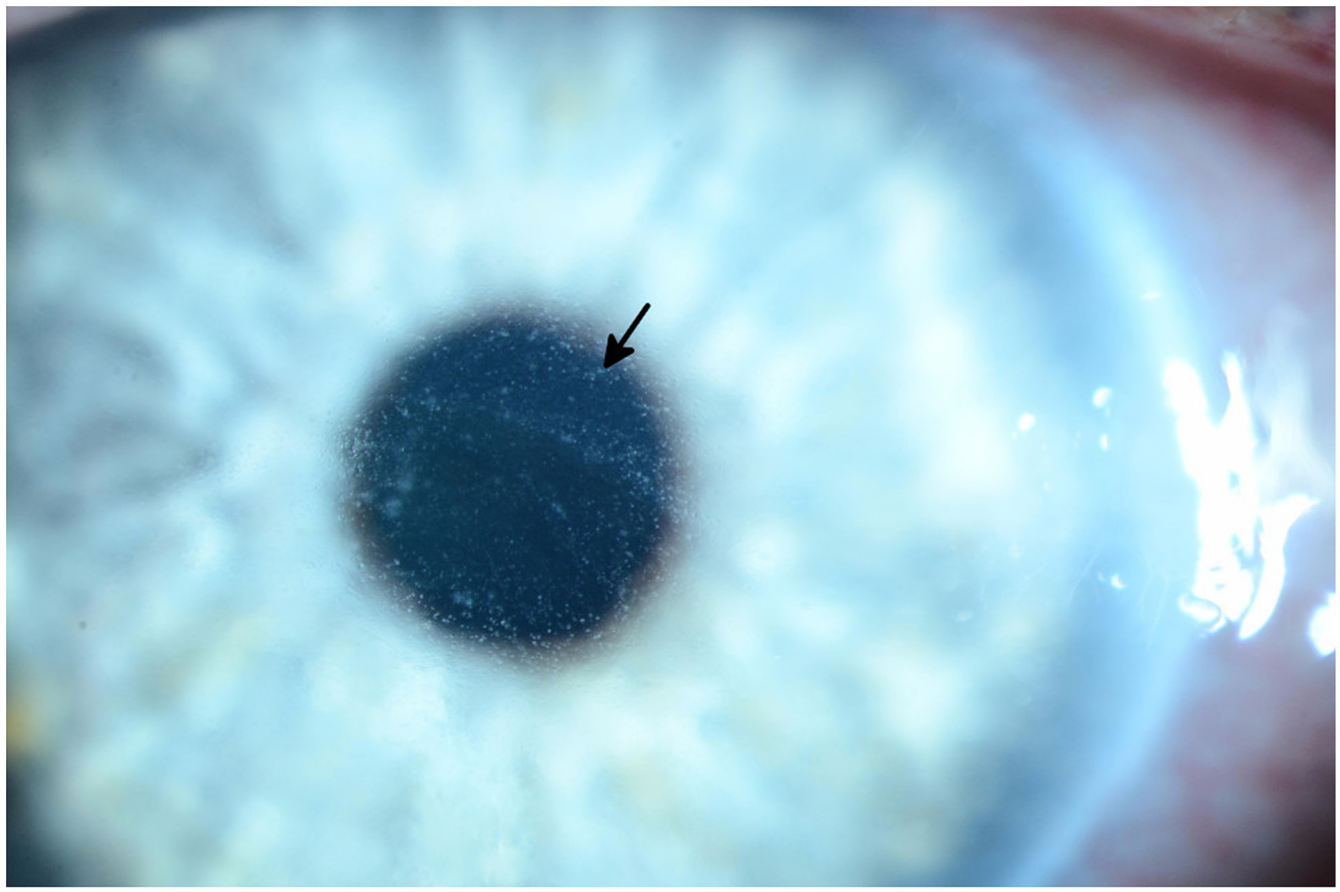
Ocular coherence tomography (OCT) shows intraretinal (black arrows) and subretinal (white arrow) fluid in a patient being treated with a MEK inhibitor who subsequently developed grade 2 retinopathy with retinal and subretinal cysts

Unlike their cytotoxic counterparts, targeted inhibitors tend to be highly specific for their molecular targets (more than 99% in most cases). As such, toxicities tend to be much more focal than systemic. Examples of this phenomenon can be seen in these data, in particular among the most severe adverse events, such as corneal ulceration, blindness, or retinal artery or vein occlusion (Figure 1), which were associated with a minority of agents. EGFR inhibitors and MEK inhibitors as a class were associated with a high proportion of severe ocular toxicities. It is known that EGFR is intimately involved in angiogenesis and wound healing [4,24]; as such, it is not surprising that the most severe ocular toxicities associated with this class of agent included complications with delayed wound healing, such as corneal ulceration. Corneal microcysts (Figure 2) have also been seen in some patients undergoing EGFR inhibitor therapy in Phase I trials. MEK inhibitors inhibit a key step in the MAPK signal transduction pathway, and an increasing body of evidence suggests that this pathway is involved in the maintenance and repair of the retina [4,12,13]. Trametinib, a MEK 1/2 inhibitor, is known to increase the risk of severe retinal issues (Figure 3), such as retinal detachment and retinal vascular occlusion.

Given the high degree of specificity that targeted agents exhibit, clinicians are better able to direct their screening of toxicity toward these specific regions of the eye that certain agents are known to affect. As such, mechanism-based screening guidelines may be appropriate for screening toxicity in targeted agents. An example taken from our management suggestions is the targeted surveillance of the retina in patients undergoing MEK inhibitor therapy. For drugs with known retinal toxicities such as trametinib, we suggest self-screening by the patient with an Amsler grid for visual field monitoring. Identification of any of these ocular symptoms merits urgent referral for additional ophthalmologic assessment. For agents known to be associated with more severe ocular toxicities (e.g., gefitinib, trametinib, vemurafenib, and ipilimumab), we suggest routine ophthalmic surveillance and baseline assessment by an ophthalmologist. A review of symptoms such as eye pain, redness, and changes in vision should be obtained by the medical oncologist at each follow-up visit.

Many of the ocular toxicities displayed by targeted agents are closely related to the drug's mechanism of action, a sentiment commonly echoed in relevant scientific literature [2–6]. However, even among drugs that target the same molecule with high specificity, variations in toxicity exist. Of note, the use of monoclonal antibodies did not typically lead to as many ocular adverse events as small-molecule agents did, even among those that shared the same mechanism of action (e.g., gefitinib and cetuximab). Ocular toxicities among monoclonal antibodies seemed to be lower in both incidence and frequency. A possible reason for this is that monoclonal antibodies are less able to permeate physiological barriers [25,26], such as the blood-brain or blood-ocular barriers, and are therefore less able to interfere directly with the delicate ocular microenvironment. The exception to this, however, is ipilimumab. Although it is believed that the antibody itself is unable to cross the blood-brain barrier, evidence suggests that activated T cells may be able to penetrate the brain [27–29]. This provides one possible explanation for why ipilimumab, among all of the monoclonal antibodies, is associated with the most severe ocular adverse events.

We attempted to conduct a comprehensive meta-analysis of targeted agents in the current literature; however, several limitations need to be acknowledged. The only agents that were included were those that were FDA-approved, so many experimental drugs were not included in this study. Most of these experimental drugs did not have sufficient study data and as such, an analysis was not feasible. A quantitative analysis was not possible due to insufficient data reported in the literature, with many agents and labels having few studies that report ocular toxicities.

Common to all retrospective analyses, variation in study quality was a limitation. Studies displayed variability and inconsistency in the reporting of ocular toxicities and ocular adverse events and in the methods of determining whether an ocular event could be attributed to the agent in question. However, the focus of the analysis was centered around phase 3 and above clinical trials validated by the FDA for quality; in this way, variability was attenuated as much as possible. Although these clinical recommendations were based on our best assessment, we recognize that many of these patients will not be receiving targeted therapy for a prolonged period and that accordingly, many of our screening recommendations may not apply in this therapeutic setting.

The CTCAE grading scale may be limited in its applications to ocular toxicity. For instance, the definition of a grade 4 adverse event, according to the CTCAE, is an event with “life-threatening consequences,” which is atypical of nearly all ocular adverse events. These limitations will be addressed in the upcoming version of the CTCAE.

**Figure 4:**
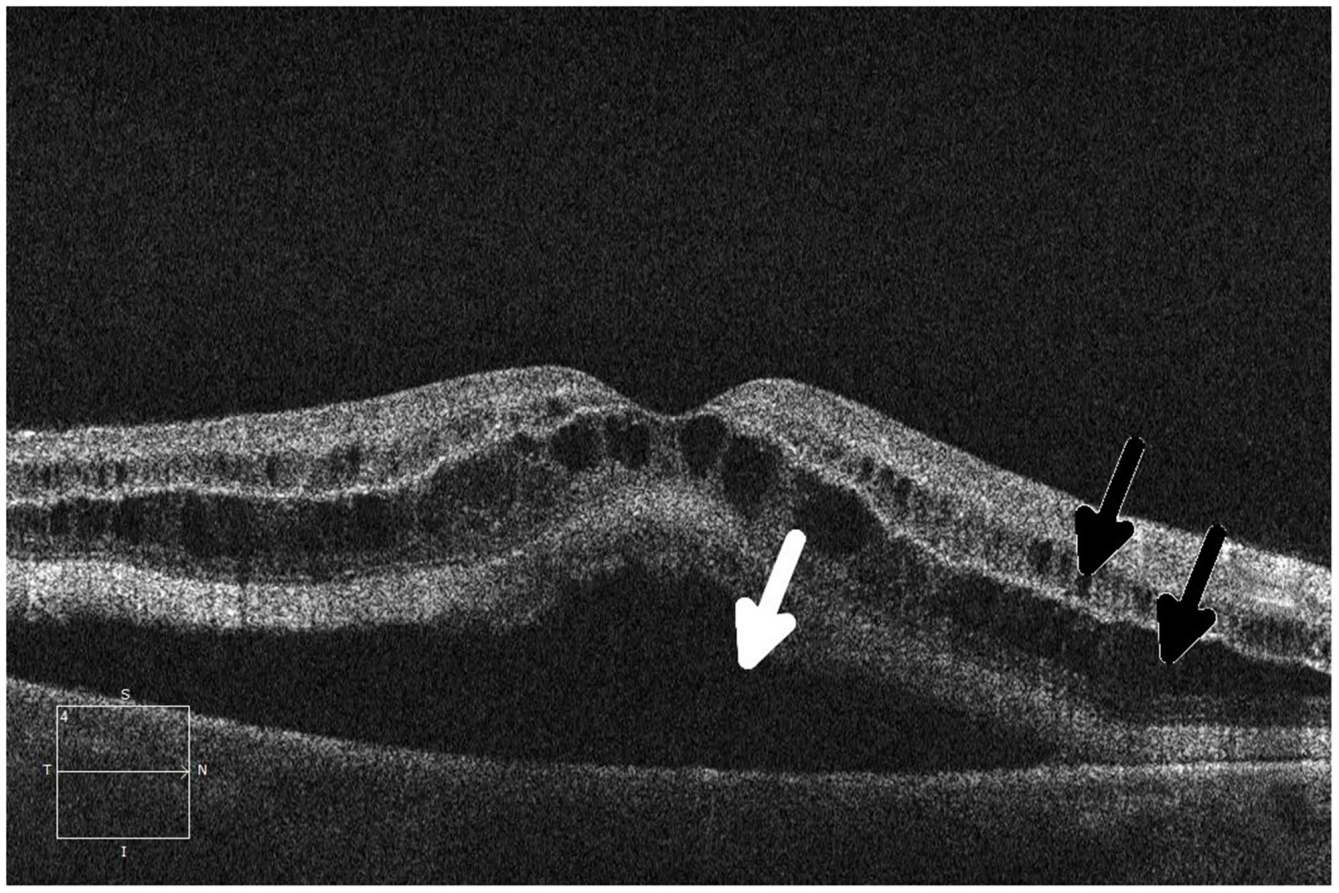
Ocular coherence tomography (OCT) displays a perpendicular cut through the retina in a patient with metastatic melanoma undergoing therapy with a small-molecule ERK inhibitor, demonstrating central serous retinopathy with subretinal fluid buildup (arrows)

Studies referenced by FDA labels provide one perspective of toxicity incidence in the general population; however, all labels acknowledge that because clinical trials are conducted under widely varying conditions, adverse reaction rates observed in the clinical trials of a drug cannot be directly compared with rates in clinical trials of another drug and may not reflect the rates observed in practice. Preliminary data are already beginning to show rates of ocular toxicity that are higher than those reported in the labels. One such example of this is with the incidence of toxicity seen in MEK inhibitors. Preliminary data show that the incidence of retinal pigment epithelial detachment with or without central serous retinopathy (Figure 4) occurred in 30 of 94 patients (31.9%) across six phase 1 clinical trials. This is a substantial increase from the reported figure (0.8%).

As evidenced by our data, ocular adverse events are an increasingly common concern in treatment with targeted anticancer agents. We hope that this study will set the stage for further specific recommendations for the screening and management of ocular toxicities in this entire class of medications. With the increased incidence of ocular toxicities, assessment by and involvement of an ophthalmologist in the treatment of patients receiving agents known to cause ocular events is merited. As the landscape of oncologic management and adverse events changes with advancing therapy, a more multidisciplinary approach to the treatment of patients with cancer is a reasonable recommendation.

## MATERIALS AND METHODS

### Drug retrieval

**Figure 5:**
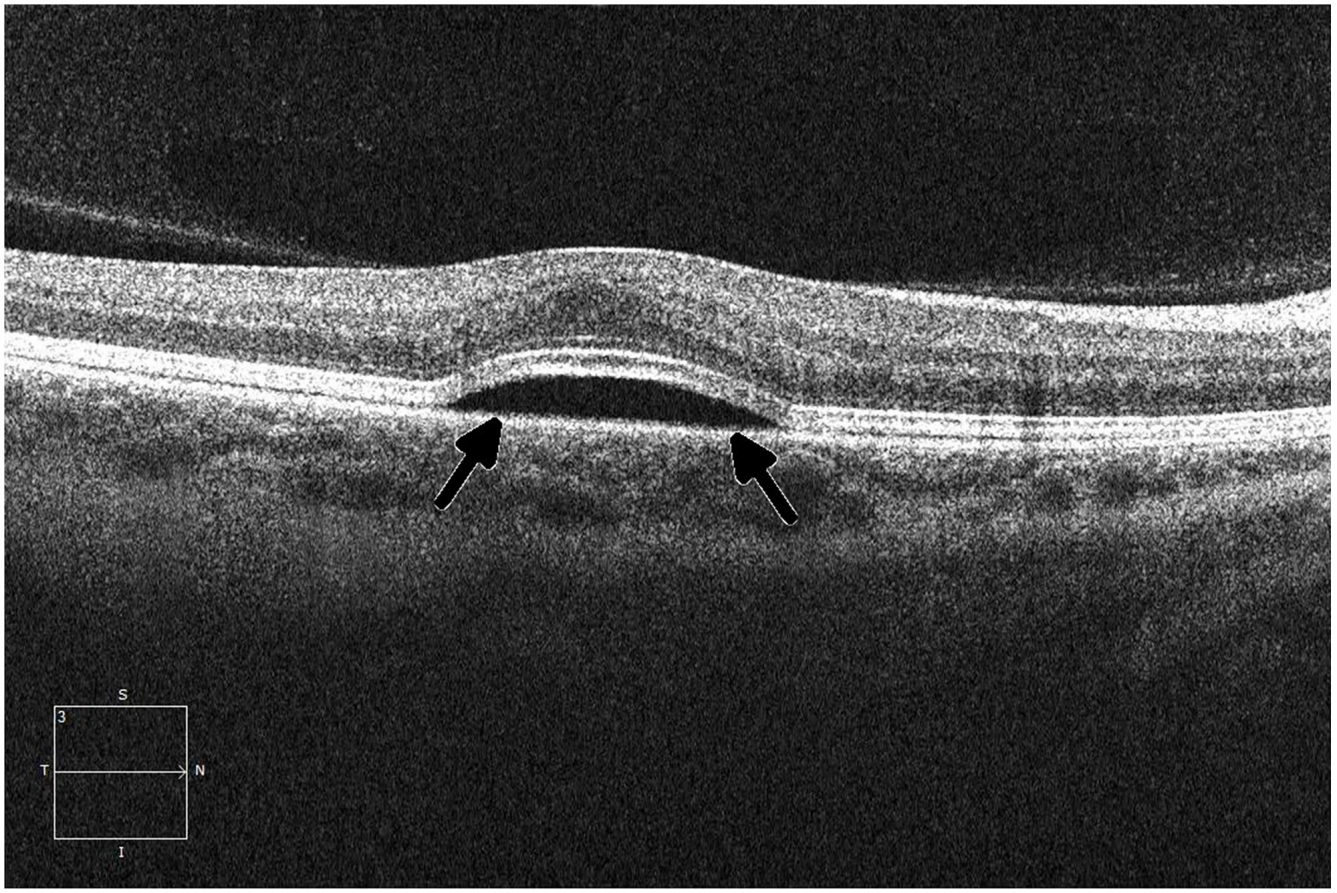
Diagram for the inclusion of anticancer agents that were analyzed All FDA-approved cancer-related agents were screened between January 1, 1998, and March 14, 2015. All non-chemotherapeutic agents, duplicate agents, and cytotoxic agents were excluded. FDA labels were retrieved for the remaining agents, and all agents that displayed evidence of ocular adverse events were included in the study. A total of 16 agents (4 monoclonal antibodies and 12 small-molecule targeted inhibitors) were initially included in the study. Four agents (bortezomib, pertuzumab, dabrafenib, and idelalisib) were associated with minor ocular adverse events according to the FDA label, but no evidence of ocular toxicity was evident upon an independent survey of the literature; these agents were therefore excluded.

The CenterWatch database of U.S. Food and Drug Administration (FDA)-approved oncologic agents was reviewed by two independent authors (C. F. and J. L.) for potential candidate agents. 138 agents that were FDA-approved between January 1, 1998, and March 14, 2015, were reviewed and screened for inclusion. We excluded all non-chemotherapeutic and duplicate agents; cytotoxic, conjugate, and immunomodulating agents; and agents with no (or insufficient) evidence of ocular adverse events (Figure 5). A review of the FDA labels for the remaining agents was performed to exclude agents for which no ocular adverse events had been reported. In addition, a simultaneous review of the literature was performed to identify any independent studies that observed these adverse events as well as any ocular adverse events observed after FDA approval of the agent. We searched Medline and Google Scholar for evidence of ocular adverse events in the remaining targeted agents. Search limits included all studies from drug inception to the present day. FDA labels for 46 targeted oncologic agents were retrieved and screened for ocular toxicity.

### Identification of ocular adverse events and study selection

Eligible studies for review included phase 1 or higher clinical trials that investigated targeted therapies as monotherapy, as well as a number of meta-analyses and pertinent reviews of the literature. Search terms included the drug name with Boolean operators AND phase NOT combination, and NOT plus. Studies were screened on the basis of relevance, patient demographics, study design, route of drug administration, and procedural integrity (i.e., randomized, double-blinded controlled trials). All studies that were included were monotherapy trials of the targeted agent. Studies that did not report ocular adverse events were excluded and were not retrieved. In addition, we excluded studies that had used small-molecule drugs in conjunction with cytotoxic therapy, those that were not monotherapy, and those that failed to include adverse events below grade 3. Clinical studies were retrieved from Medline, Google Scholar, the Cochrane database, and the NIH Clinical Trials Database. Studies searched on the NIH database were limited to those with results and were reviewed for inclusion criteria and possible retrieval based on study details provided by the sponsor institution.

All ocular adverse event frequencies and severities were identified on the basis of data from both FDA labels and independent clinical studies. When available, FDA label–referenced studies were retrieved and used to create an overview of ocular toxicities across all FDA label–referenced studies. If discrete FDA data were unavailable, data from independent studies were screened for inclusion criteria and reported.

### Study retrieval and data pooling

Two authors independently reviewed the abstracts and figures of all eligible studies. All relevant mention of ocular adverse events were noted and retrieved. Of these studies, the authors reviewed the data and methods for sufficient rigor and independently assessed for risk of bias. All studies that failed to report nominal data, failed to partition the data into discrete adverse events, or failed to report total adverse events were excluded.

Studies were reviewed according to selection criteria. Of these, only studies that reported ocular adverse events were included. Meta-analyses and review articles were reviewed and retrieved as indicated. Data on ocular adverse events, including frequency of independent events, were gathered and reported from FDA labels and selected studies. If the cohort demographics and study designs were similar, the results were pooled. From these data of ocular adverse events, severity of toxicities were separated on the basis of Common Terminology Criteria for Adverse Events v4.03 (CTCAE) grade [22].

### Drug comparison

Drugs were separated into two classes: small-molecule targeted inhibitors and monoclonal antibodies. Data from the two groups of targeted therapies were analyzed to determine significant differences between them. Specific parameters examined included overall frequency of ocular adverse events, frequency of events graded at least CTCAE grade 3, the most frequent ocular adverse events in each class, and the percentage of drugs that were associated with ocular adverse events in each class. Significant findings were then re-examined between with agents that were matched by mechanism of action. Percentages of drugs per class causing ocular events were calculated as total number of agents with known ocular adverse events divided by total number of agents in the group.

### Formulation of clinical recommendations

Known ocular adverse events were screened for most pertinent events. Ocular adverse events were included on the basis of the following characteristics: Incidence and frequency of the event, acuteness and aggressiveness of onset, severity of the event, and irreversibility of the event. Next, existing guidelines were retrieved for drugs that have well-documented ocular adverse events (e.g., hydroxychloroquine, ethambutol). A review of the literature was performed to identify relevant meta-analyses, randomized controlled trials, reviews, case-control or cohort studies, and case reports or case-series. Interventions and recommendations for rare or unique cases were identified in case studies. Recommendations were formulated with the input of an expert consultant with extensive expertise in dealing with ocular toxicities (DSG).
