# Erratum: Ocular toxicities associated with targeted anticancer agents: an analysis of clinical data with management suggestions

**DOI:** 10.18632/oncotarget.26658

**Published:** 2019-01-29

**Authors:** Chen Fu, Dan S. Gombos, Jared Lee, Goldy C. George, Kenneth Hess, Andrew Whyte, David S. Hong

**Affiliations:** ^1^ Department of Internal Medicine, New York University Langone Medical Center, NY 10016, USA; ^2^ Department of Internal Medicine, Baylor College of Medicine, TX 77030, USA; ^3^ Department of Phase I Clinical Trials, The University of Texas MD Anderson Cancer Center, TX 77030, USA; ^4^ Department of Biostatistics, The University of Texas MD Anderson Cancer Center, TX 77030, USA; ^5^ Department of Head and Neck Surgery, Division of Ophthalmology, The University of Texas MD Anderson Cancer Center, TX 77030, USA

**Figure 1:**
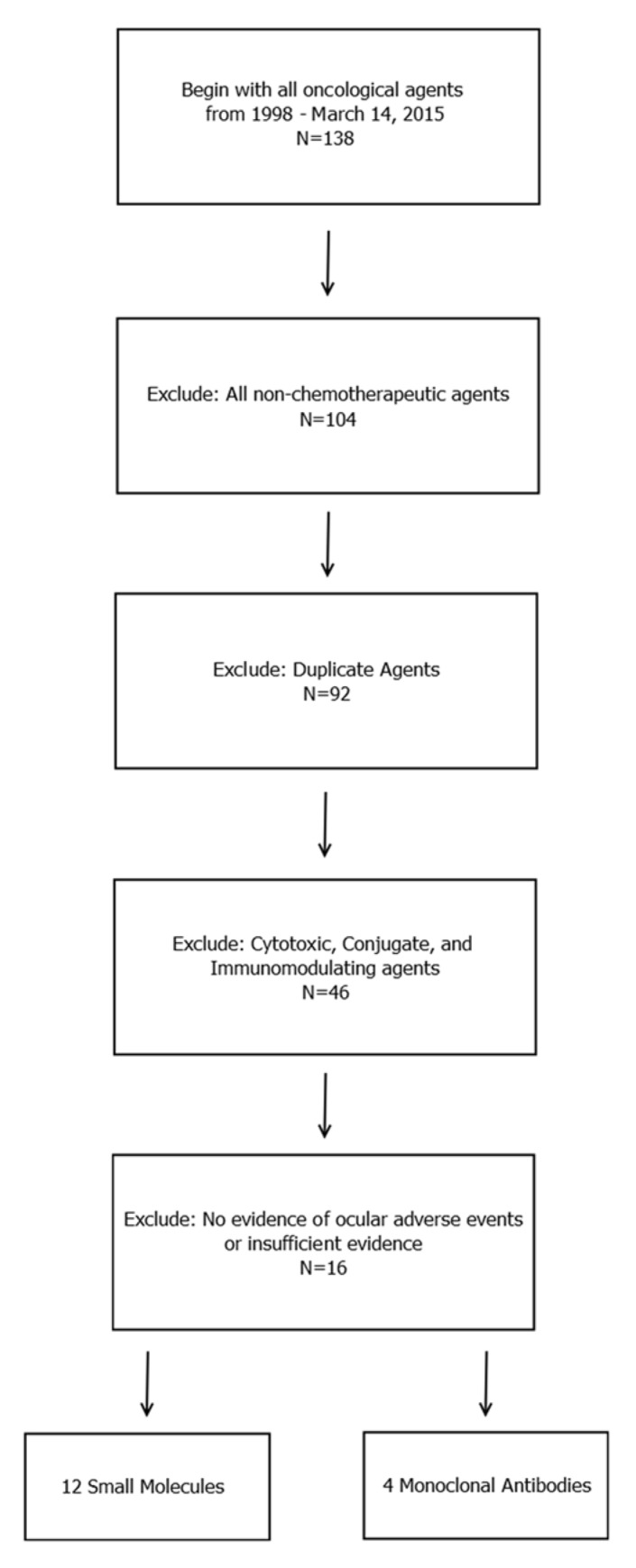
Diagram for the inclusion of anticancer agents that were analyzed All FDA-approved cancer-related agents were screened between January 1, 1998, and March 14, 2015. All non-chemotherapeutic agents, duplicate agents, and cytotoxic agents were excluded. FDA labels were retrieved for the remaining agents, and all agents that displayed evidence of ocular adverse events were included in the study. A total of 16 agents (4 monoclonal antibodies and 12 small-molecule targeted inhibitors) were initially included in the study. Four agents (bortezomib, pertuzumab, dabrafenib, and idelalisib) were associated with minor ocular adverse events according to the FDA label, but no evidence of ocular toxicity was evident upon an independent survey of the literature; these agents were therefore excluded.

**Figure 2:**
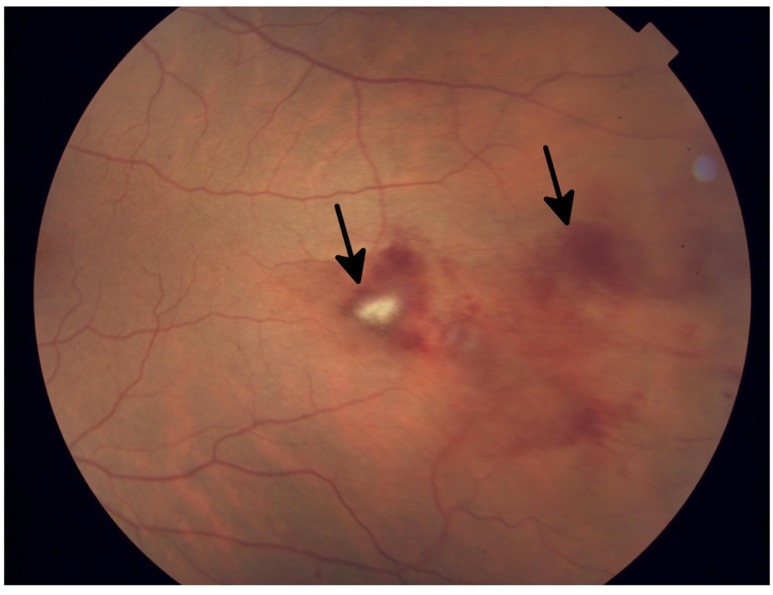
Fundus photograph of the left eye in a patient who developed grade 1 branch vein occlusion while undergoing MEK inhibitor therapy Arrows denote dot blot hemorrhages in the fundus, consistent with occlusion.

**Figure 3:**
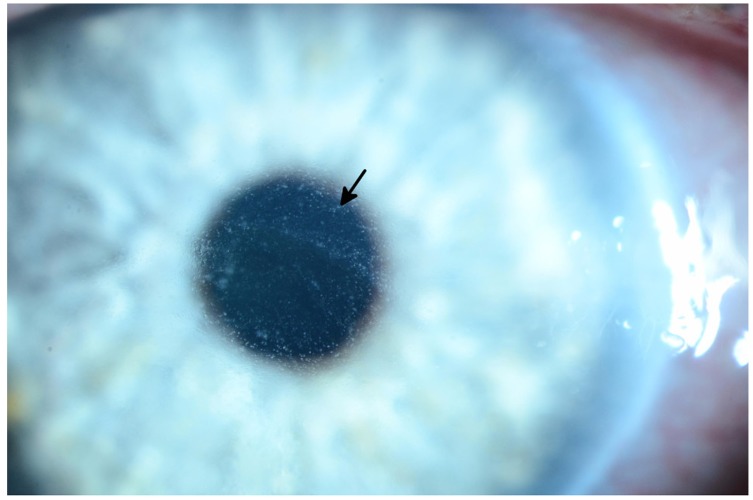
Slit lamp photograph of grade 3 diffuse microcystic changes (arrow) in a patient undergoing treatment with an EGFR inhibitor The patient subsequently developed ocular hypertension due to the topical steroid used to treat the microcysts.

**Figure 4:**
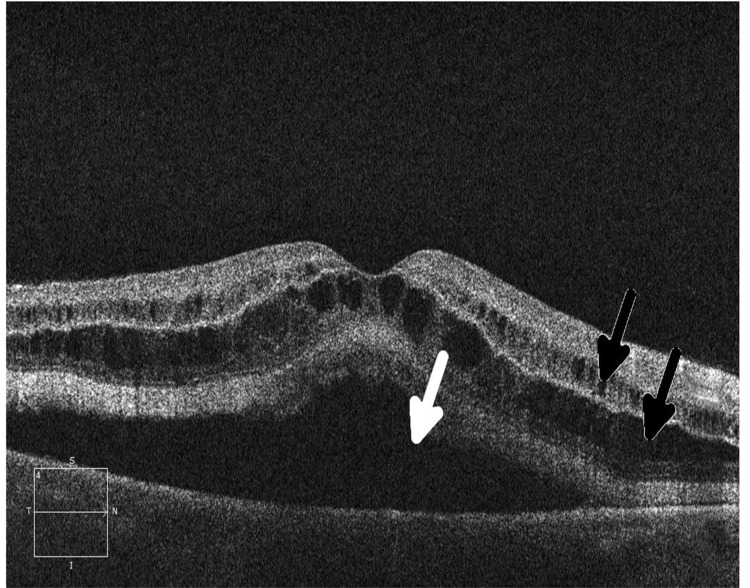
Ocular coherence tomography (OCT) shows intraretinal (black arrows) and subretinal (white arrow) fluid in a patient being treated with a MEK inhibitor who subsequently developed grade 2 retinopathy with retinal and subretinal cysts

**Figure 5:**
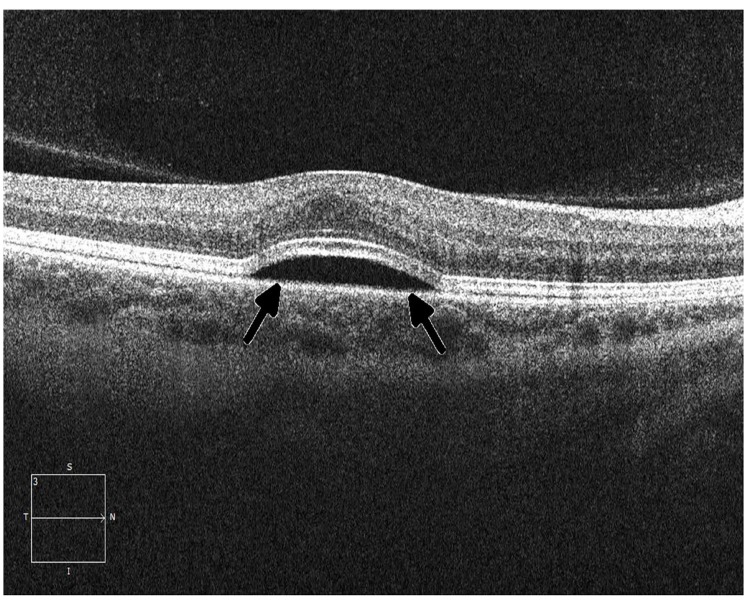
Ocular coherence tomography (OCT) displays a perpendicular cut through the retina in a patient with metastatic melanoma undergoing therapy with a small-molecule ERK inhibitor, demonstrating central serous retinopathy with subretinal fluid buildup (arrows)

**This article has been corrected:** Due to a production error, the figures in this paper were mislabeled. The correct figure legends are given below:

Original article: Oncotarget. 2017; 8:58709-58727. https://doi.org/10.18632/oncotarget.17634

